# Association of shift work with body weight, stress, sleep, and dietary intake in prison officers and firefighters

**DOI:** 10.1007/s00394-026-03923-x

**Published:** 2026-03-05

**Authors:** Leonida N. Mosomi, Claire L. Fyfe, Graham W. Horgan, Kim Giles, Daryl B. O’Connor, Baukje de Roos, Alexandra M. Johnstone

**Affiliations:** 1 The Rowett Institute, University of Aberdeen, Aberdeen, AB25 2ZD UK; 2 Biomathematics and Statistics Scotland, Foresterhill Road, Aberdeen, AB25 2ZD UK; 3 Laboratory for Stress and Health Research (STARlab), University of Leeds, Leeds, LS2 9JT UK

**Keywords:** Prison officers, Firefighters, Shift work, Overweight and obesity, Dietary patterns, Sleep, Stress

## Abstract

**Purpose:**

We explored how shift work in UK prison officers and firefighters is associated with body composition, stress, and sleep, and with the quantity, quality, and timing of dietary intake whilst on-shift and off-shift.

**Methods:**

Secondary analysis of cross-sectional data from an intensive 7 day study on anthropometry, stress, sleeping patterns and dietary intake, obtained in 22 prison officers and 51 firefighters (both male and female), taking part in the neurobiology of food addiction and stress (NeuroFAST) study.

**Results:**

Mean age was 37.4 ± 7.23 years, while 78% of prison officers and 61% of firefighters were classified as living with either overweight or obesity. Overall, daily energy and total fat intakes were within recommended ranges. However, carbohydrate and dietary fibre intakes were below, while saturated fat and salt intakes exceeded, recommended amounts for health. Prison officers had a significantly lower frequency of breakfast and dinner occasions when on-shift, whereas firefighters had a significantly lower frequency of all eating occasions when on-shift, than when off-shift (all* p* < 0.001). In prison officers, intake of energy and sugar was significantly higher when off-shift (*p* = 0.001 and *p* = 0.002, respectively). Both prison officers and firefighters had higher stress scores during shift days than on non-shift days (*p* = 0.007 and *p* < 0.001 respectively). Both groups had longer sleep durations on non-shift days than on shift days (all *p* < 0.001).

**Conclusion:**

Prison officers and firefighters had a high prevalence of overweight and obesity and their dietary patterns, and timing of eating occasions, especially when off-shift, may increase risk of metabolic disease.

**Supplementary Information:**

The online version contains supplementary material available at 10.1007/s00394-026-03923-x.

## Introduction

Shift work accounts for a large proportion of workers globally [1]. In 2018, 14.1% of the UK workforce worked in shifts and approximately 165,000 people were employed in shift work in the protective services including prison, fire, and police service [2]. Shift work requires workers to be on duty at unconventional hours, and as a consequence, the sleep/wake cycle is functioning against the natural biological diurnal cycle [3]. Shift work increases the risk of metabolic syndrome [4, 5], some cancers [6, 7], mental health issues [8, 9], and altering the quality and/or duration of sleep [10, 11]. For example, one observational study revealed that a higher proportion of shift workers (30.5%) had higher ratios of serum triglycerides to high density lipoproteins cholesterol (TG/HDL-C ratio) > 3.5 compared to 8.6% of daytime workers [12] that is a predictor of insulin resistance. Shift work may also lead to work-related and psychosocial stress, which itself is associated with an increased risk of metabolic syndrome [13], and some cancers [14]. Shift work affects sleep patterns [15], including short sleep duration, poor sleep quality and altered sleep timings, which are likely contributors to adverse health outcomes [9, 16].

Shift work may also indirectly affect health by changing the timing of eating occasions [17, 18]. Workers on night shifts tend to consume more calories during evening time [19, 20], thus affecting the daily intake of energy [21]. Eating late and during the night may be detrimental to metabolic health [18], with epidemiological and cross-sectional studies suggesting a link with obesity [22, 23], elevated levels of low-density lipoproteins (LDL) and total cholesterol [24], reduced glucose tolerance [25], and reduced insulin sensitivity [26].

Working shifts has also been associated with a higher consumption of snacks rather than meals, often resulting in lower dietary fibre and carbohydrate intake and higher consumption of saturated fat and salt [21, 27]. These dietary patterns are particularly evident among firefighters and prison officers, who frequently rely on convenience foods during long or irregular shifts. In addition to poor dietary habits, shift workers are more likely to engage in detrimental lifestyle habits such as smoking and binge drinking [28, 29]. These combined factors contribute to an increased risk of obesity, cardiovascular disease, and metabolic disorders, which are more prevalent in shift-working populations [27, 29]. Shift routines therefore have the potential to affect meal patterns and food choice [30, 31]. Peripheral clocks in metabolic tissues are synchronised by behavioural cues, particularly food intake, which acts as a potent ‘Zeitgeber’ (time giver cue) for the liver and gut. During shift work, mistimed eating during the night cycle can uncouple these peripheral clocks from the centrally regulated circadian clock, leading to internal circadian misalignment*.* This highlights the importance of chrono-nutrition, whereby aligning the timing of food intake with circadian biology helps maintain synchrony between central and peripheral clocks, supporting metabolic health and reducing cardiometabolic risk.

Shift patterns, and challenging working conditions may negatively affect the health of prison officers and firefighters [32–34]. Findings from cross-sectional studies revealed higher rates of hypertension among female correctional officers than in other public workforces [35], and there is a higher prevalence of hypertension in firefighters than in the general population [36, 37]. The prevalence of overweight and obesity in prison officers and firefighters was also higher than in other workers [33, 38, 39]. However, thus far most studies have not addressed the complex interactions between overweight and obesity, stress and sleeping patterns, and the quality and timing of food and meal consumption. This cross-sectional observational study aimed to assess how shift work schedules in prison officers and firefighters in the UK are associated with body composition, stress, and sleep, but also with the quantity, quality, and timing of dietary intake.

## Methods

This was a secondary analysis of observational data collected for a study on stress and eating behaviour conducted in Scotland [40], in the NeuroFAST (neurobiology of food addiction and stress) study, part of a larger multicentre NeuroFAST project, conducted in seven different countries: the UK, Sweden, the Netherlands, Italy, Germany, Hungary and Spain [41]. The research protocol was approved by the North of Scotland Research Ethics Committee (reference no: 10/S0801/66) in alignment with the Declaration of Helsinki agreement on ethical principles for medical research involving human subjects. All participants gave written consent and were free to withdraw from the study at any time.

### Participants

The NeuroFAST study recruited 422 participants from different public sector workforces, including prison officers working in the Scottish Prisons Service, and firefighters working in the Scottish Fire and Rescue Service. Participants were both male and female, and above 18 years of age. Participants who were pregnant, had a history of an existing psychiatric condition (and on treatment for the last six months), or those that had a change in medication in the last three months for a pre-existing condition (for example, use of insulin or statins), were excluded. The current study used a subset of data from the original study cohort. A total of 74 participants who worked variable shift patterns were included in the subset: 22 prison officers (15 males, 7 females) and 52 firefighters (45 males, 7 females).

### Study design

All measurements on the NeuroFAST study visits were conducted in designated rooms at the participants’ workplace. The study design can be accessed in Supporting Information -Online Resource 1.

In summary, at the baseline visit, demographic information (age and gender) and anthropometric measurements (weight, height, waist and hip circumference, estimation of abdominal and visceral fat) were recorded. During seven consecutive days, participants were asked to record an ad libitum food diary using food weighing scales, to monitor their physical activity using an Actigraph triaxial accelerometer that detects motion (Actigraph LLC, Pensacola, USA), and to complete a series of self-reported questionnaires including the Daily Hassles Scale to report on stress, shift-type, sleep, work duration, the Hourly Visual Analogue Scale (VAS) to assess hunger, appetite, stress, and an End of Day questionnaire to monitor perceived hunger, and appetite. After seven days, during the final visit, participants completed the Depression, Anxiety and Stress Scale (DASS-_21_), and submitted the diaries and questionnaires. More details on each of the study assessments are in the paragraphs below.

### Anthropometric measurements

Participants’ weights were measured using a portable digital human weighing scale (SECA, Hamburg, Germany) in light clothing, and the weight rounded off to the nearest 0.1 kg. A standard one kilogram was deducted from each participant’s weight to account for the weight of their clothing. Height measurements were taken and recorded to the nearest 0.1 cm using a portable Stadiometer (Model 213, SECA, Hamburg, Germany) ensuring that the participant stood in an erect position, facing straight ahead, with the torso and the back of the heels against the stadiometer and, the feet apart and firm on the base. Body Mass Index (BMI, kg/m^2^) was calculated using the corrected weight and height, and the results categorized according to the World Health Organisation (WHO) classifications: normal weight (18.5–24.9 kg/m^2^), overweight (25.0–29.9 kg/m^2^), and obese (> 30.0 kg/m^2^). Waist and hip circumferences were measured using a metal tape measure to the nearest 0.1 cm and used to calculate the waist to hip ratio. The ratio results were classified using the WHO cut-off points for risk of metabolic complications (waist circumference: increased risk > 94 cm for males and > 80 cm for females; substantially increased risk > 102 cm for males and > 88 cm for females; waist-to-hip ratio: substantially increased risk ≥ 0.90 for males and ≥ 0.85 for females). Measurements for abdominal and visceral fat (%) was estimated through a Bioelectrical Impedance Analysis (AB 140 ViScan, Tanita Corporation, Tokyo, Japan) whilst the participant was lying in a supine position. An infrared beam was projected over the waist at the umbilical saggital plane, detected by two infrared sensors on either side of the base unit. Impedance was then measured by ViScan, which is essentially a tetrapolar impedance method involving two pairs of injecting and sensing electrodes (basically a wireless measurement ‘belt’) placed directly on the skin at the umbilicus in the saggital plane. ViScan abdominal body composition values are derived from extrapolation of impedance measures (at 6.25 and 50 kHz) using inbuilt software. Abdominal body composition values are subdivided into total abdominal or trunk adiposity (that is, intra-abdominal adipose tissue [IATT] + subcutaneous adipose tissue [SAAT]), expressed as percentage trunk fat, and IAAT, which is expressed as ‘visceral fat’.

### Stress

Stress was assessed using an hourly visual analogue scale (VAS), a daily hassles scale and a one-off Depression Anxiety and Stress Scale (DASS-_21_). The VAS was completed by asking the participants to mark how stressed they felt during each waking hour, on a 100 mm scale [42]. The scores from the VAS were used to track temporal changes of stress and its relation to dietary intake. Throughout the seven days of the study, participants completed the daily hassles scale with a short description of events that they had perceived to be stressful during the day (stressors) as well as the time of the occurrence, rating the intensity of each stressor using a four-point scale (e.g., 0–1 being not stressful, 2 being stressful, 3 being very stressful, and 4 being extremely stressful). The stressors were later coded as ‘ego-threatening, interpersonal, work related, physical or environmental’ and a mean severity score was calculated for each stressor as well as the total for all days [43]. The DASS-_21_ questionnaire was completed at the end of the study to assess stress, as one of the negative emotional states, retrospectively for the previous seven days. Scores from the DASS-_21_ were then multiplied by two to calculate and interpret the final score, with normal being a score of 0–14, mildly stressed being a score of 15–18, moderately stressed being a score of 19–25, severely stressed being a score of 26–33 and extremely stressed being a score of above 34.

### Sleep duration

Sleep duration was assessed using the Actigraph triaxial accelerometer, and an activity log indicating the time that the Actigraph was applied and removed during sleep. Participants were also asked to record the number of hours they had slept the previous night, and how many hours they had spent at work that day, in the Daily Hassles questionnaire. Extracted data were analysed over a 24-h cycle beginning at 5:00 AM (designated as hour 1) and ending at 4:00 AM (hour 24) to align with participants’ diurnal rhythms and typical wake times.

### Dietary assessment

Participants were asked to complete a 7-day ad libitum food diary, by recording time, weight, eating occasion, and cooking methods of all food and drinks consumed, as eaten. Calibrated kitchen scales (Disc Electronic Kitchen Scale 1036, Salter Housewares, UK) were provided to weigh the food consumed and note all leftovers. For foods participants were unable to weigh, they would indicate the household food measure that was used, for example, a mug of tea, a levelled teaspoon, as well as attaching any food labels they had for convenience meals. The food diary contained spaces to input recipes. If no weights or portion sizes were recorded, then standard portion sizes were used [44].

The 7-day food diaries were inputted using WinDiets software, using an electronic version of McCance & Widdowson nutrition database (2015). When the participant gave information on a composite meal, for example, a home-made recipe, we used the individual ingredients for analysis. Alternatively, if the composite meal (e.g. sandwich or lasagne ready meal) was shop bought, we would access the online nutrition information from the food retailer. If no information was provided beyond a description of the meal, we used the nearest composition in the McCance & Widdowson database (2015). Data were checked, customized for time of food intake, and coded by shift and eating occasion for further analysis using Microsoft Excel spreadsheets. The participants supplied details of what their shift pattern was for the 7 days of the study, eating occasions could therefore be coded as on or off shift. For the purposes of dietary analysis, 'on-shift' periods were defined as any time during designated working hours on duty days, including both active duty and standby time, during which food and beverage consumption was recorded as occurring while on duty. Conversely, 'off-shift' periods encompassed all food intake outside of these working hours, including meals and snacks consumed on days off. Eating occasions were defined by the participants as either a breakfast, lunch, dinner or snack which were then analysed for frequency according to the number of times each meal type was consumed by the participants over the 7 days. Mean daily energy and nutrient intakes from all eating occasions were calculated. The data was also analysed according to the time of day of the eating occasions and whether they were on or off shift. The ratio of energy intake (EI) to basal metabolic rate (BMR) was calculated to check for under and over reporting. Basal metabolic rate was calculated using the Schofield equation [45].

### Hunger and appetite

Hunger and appetite were recorded daily using the hourly 100 mm paper VAS, during waking hours. An End-of-Day Questionnaire was completed daily, also in a VAS style, to assess how hungry or full the participants had felt on average during each day.

### Data analysis

Descriptive statistical analysis was performed in SPSS Version 28.0 (IBM SPSS Statistics for Windows, Version 28.0.). Normality for all the data was checked visually using histogram plots and through normality tests (Kolmogorov–Smirnov (K-S) with Lilliefors corrected K-S test, and Shapiro–Wilk tests. Data analysis was performed separately for prison officers and firefighters—whilst both shift workers, their shift conditions and place of work are markedly different and therefore results may not necessarily be comparable. Differences in dietary intakes between the shifts and between meals and snacks were measured using a paired t-test (for normally distributed data) and Wilcoxon test (for skewed data). Proportions of frequency for eating occasions according to shifts were assessed using Wilcoxon test. Pearson’s correlations were used to assess the associations between stress and dietary intake. Using an ordinary least squares (OLS) regression model, we examined the relationship between objectively measured sleep duration (via actigraphy) and three predictors: waist circumference, age, and occupational group (prison officers versus firefighters). As there were a large number of p-values calculated, both for differences and for correlations, we applied the Benjamini–Hochberg procedure [46] with the family-wise error rate set to 0.05. For the p-values obtained to test differences, this indicated that all p-values less than 0.013 should be accepted. We have therefore concentrated our discussion on differences where *p* < 0.01. When applied to the set of p-values obtained from correlations, the procedure indicated that only the strongest p-value was significant.

## Results

Data from 74 participants (22 prison officers and 52 firefighters) were considered for analysis. However, data from one firefighter was excluded as the average energy intake in this participant was more than 6,000 kilocalories per day (EI:BMR ratio = 3.10). Therefore, data from 73 participants were analysed for this study.

### Body composition, stress, and sleep

Main age, anthropometric characteristics, and body composition of the prison officers and firefighters are presented in Table 1. The majority of prison officers and firefighters were classified as overweight (77.3% and 60.7%, respectively), including 31.8% and 7.84%, respectively, who were classified as obese. Additionally, 59.1% of prison officers and 27.5% of firefighters were classified as having increased or substantially increased risk of metabolic complications as assessed by waist circumference.

**Table 1:** Main demographic and body composition characteristics for prison officers and firefighters

	Average in Scottish population^#^	Total study population (n = 73)	Prison officers (n = 22)	Firefighters (n = 51)
Age (yrs)	**–**	37.4 ± 7.23	38.7 ± 7.85	36.9 ± 6.96
Height (m)	1.69	1.76 ± 0.07	1.75 ± 0.08	1.77 ± 0.07
Weight (kg)	79.6	82.7 ± 13.2	86.7 ± 15.6	80.9 ± 11.8
BMI (kg/m^2^)^a^	27.9	26.6 ± 3.51	28.2 ± 4.28	25.9 ± 2.90
Normal weight n (%)	33%	25 (34.2)	5 (22.7)	20 (39.2)
Overweight n (%)	37%	37 (50.7)	10 (45.5)	27 (52.9)
Obese n (%)	29%	11 (15.1)	7 (31.8)	4 (7.84)
Trunk body fat (%)^b^	–	28.4 ± 8.58	34.6 ± 8.83	25.8 ± 7.03
Visceral fat (%)^c^	–	11.2 ± 5.62	14.0 ± 7.37	10.0 ± 4.21
Waist circumference (cm)^c^	93.2	89.2 ± 10.5	93.7 ± 13.0	87.2 ± 8.62
Male (n = 59)^c,d^	97.2	91.5 ± 8.49	–	–
Female (n = 14)^c,d^	87.9	79.2 ± 12.5	–	–
Increased risk^d^ n (%)	–	18 (24.7)	6 (27.3)	12 (23.5)
Substantially increased risk^d^, n (%)	–	9 (12.3)	7 (31.8)	2 (3.92)
Total risk^e^^,f^, n (%)		27 (37.0)	13 (59.1)	14 (27.5)
Hip circumference (cm)	–	103 ± 7.67	108 ± 9.67	101 ± 5.40
Waist/hip ratio^c^	–	0.86 ± 0.07	0.86 ± 0.08	0.86 ± 0.06
Male (n = 59)^c,d^	–	0.88 ± 0.06	–	–
Female (n = 14)^c,d^	–	0.78 ± 0.05	–	–
Increased riskf, n (%)	–	24 (32.9)	7 (31.8)	17 (33.3)

Values represent means ± SD and n (percentages)

^#^2019 Scottish Health Survey [43]

^a^BMI categorized according to the World Health Organisation (WHO) classifications: normal weight (18.5 to 24.9 kg/m^2^), overweight (25 to 29.9 kg/m^2^), and obese (above 30 kg/m^2^)

^b^Trunk and visceral fat measured by ViScan (Tanita Corporation), a form of bioimpedance measurement of

^c^Data presented for all participants, irrespective of the WHO cut-off points

^d^Data by gender for Prison officers (M = 15, F = 7) and Firefighters (M = 44, F = 7) not presented due to small sample sizes

^e^WHO cut-off points and risk of metabolic complications [waist circumference: increased risk > 94 cm (male), > 80 cm (female); substantially increased risk > 102 cm (male), > 88 cm (female); waist to hip ratio: increased risk ≥ 0.90 cm (male), ≥ 0.85 cm (female)]

^f^Number of participants with waist circumferences above both the increased, and substantially increased, risk WHO cut-off points

The DASS-_21_ stress scores were within the normal range (0–14), for both prison officers and the firefighters (9.91 ± 8.59 and 6.04 ± 6.34 respectively). All stressors from the daily hassles questionnaire had a severity score greater than 2, thus were rated as stressful (Table 2).

**Table 2:** Indicators of stress as assessed by the DASS-_21_, the daily hassles questionnaires, and the Visual Analogue Scale (VAS), over a period of 7 days

	Total study population (n = 73)	Prison officers (n = 22)	Firefighters (n = 51)
*DASS-*_*21*_* questionnaire*^*a*^			
Stress score	7.21 ± 7.26	9.91 ± 8.59	6.04 ± 6.34
*Daily hassles questionnaire*^*b*^			
Ego-threatening	2.17 ± 1.17	2.00 ± 0.00	2.25 ± 1.50
Interpersonal	2.40 ± 0.91	2.64 ± 0.78	2.31 ± 0.97
Work-related	2.36 ± 0.80	2.33 ± 0.81	2.38 ± 0.81
Physical	2.67 ± 1.15	3.13 ± 0.77	2.38 ± 1.30
Environmental	2.44 ± 0.70	2.57 ± 0.84	2.39 ± 0.66
Total	2.55 ± 0.73	2.71 ± 0.75	2.47 ± 0.72
*VAS scale (0-100 mm)*			
Stress score	7.50 (0–100)	8.30 (0–94.7)	7.30 (0–100)

Values represent mean ± SD and median (min–max)

^a^Cut-off score: normal 0–14, mild 15–18, moderate 19–25, severe 26–33, extremely severe 34 +

^*b*^Cut-off score: 0–1 not stressful, 2 stressful, 3 very stressful, 4 extremely stressful. DASS, Depression Anxiety Stress Scales

During the seven days, prison officers spent an average (± SD) of 115 ± 5 h awake and 53 ± 5 h asleep, whereas firefighters spent an average (± SD) of 107 ± 10 h awake and 61 ± 10 h asleep, as calculated from the Actigraph data. Sleep duration from the Daily Hassles questionnaire showed that prison officers and firefighters spent an average (± SD) of 53 ± 5 and 49 ± 6 h asleep during the seven days respectively.

### Quantity, quality, and timing of dietary intake

The intake of nutrients in study participants was compared with the UK dietary reference values (DRVs) [47] to evaluate diet quality. Generally, the daily intake of energy and total fat in both prison officers and firefighters was within recommendations. Carbohydrate and dietary fibre intake were lower than recommended, whereas the intake of saturated fat and salt (g) was higher than recommended, in both prison officers and firefighters (Table 3). However, the ratio of energy intake to basal metabolic rate (BMR) suggested that prison officers may have underreported their dietary intake.

**Table 3:** Average daily intake of energy and nutrients in prison officers and firefighters over 7 days

	Dietary reference values (DRV)^a^	Total population (n = 73)	Prison officers (n = 22)	Firefighters (n = 51)
Energy (kcals/d)	–	2,310 ± 625	1,840 ± 471	2,510 ± 578
Male (n = 59)^b^	2,500	2,430 ± 609	–	–
Female (n = 14)^b^	2,000	1,790 ± 395	–	–
BMR (kcal/d)^c^	–	1,790 ± 221	1,800 ± 260	1,790 ± 204
Male (n = 59)	–	1,880 ± 126	–	–
Female (n = 14)	–	1,430 ± 145	–	–
Energy intake:BMR	–	1.30 ± 0.34	1.04 ± 0.30	1.41 ± 0.30
Male (n = 59)	–	1.30 ± 0.35	–	–
Female (n = 14)	–	1.28 ± 0.33	–	–
Fat (g/d)	–	86.9 ± 27.2	65.4 ± 22.3	96.1 ± 23.8
Male (n = 59)	97	90.7 ± 26.8	–	–
Female (n = 14)	78	70.8 ± 23.3	–	–
Saturated fat (g/d)	–	32.5 ± 11.4	24.7 ± 10.2	35.9 ± 10.3
Male (n = 59)	31	34.0 ± 11.5	–	–
Female (n = 14)	24	26.3 ± 9.18	–	–
Protein (g/d)	–	97.1 ± 33.3	79.1 ± 29.7	105 ± 31.9
Male (n = 59)	–	103 ± 33.6	–	–
Female (n = 14)	–	72.6 ± 17.0	–	–
Carbohydrate (g/d)	–	267 ± 85.4	211 ± 54.4	291 ± 85.5
Male (n = 59)	333	281 ± 81.7	–	–
Female (n = 14)	267	207 ± 76.3	–	–
Sugars (g)	90	97.7 ± 40.2	81.3 ± 32.7	105 ± 41.3
Male (n = 59)	–	101 ± 41.5	–	–
Female (n = 14)	–	85.4 ± 32.3	–	–
Starch (g)	135	149 ± 60.2	107 ± 36.4	167 ± 59.9
Male (n = 59)	–	161 ± 57.1	–	–
Female (n = 14)	–	98.2 ± 46.1	–	–
Fibre (g/d)	30	15.7 ± 6.36	16.2 ± 7.46	15.4 ± 5.88
Male (n = 59)	–	16.1 ± 6.56	–	–
Female (n = 14)	–	13.7 ± 5.16	–	–
Alcohol (g/d)	–	6.81 (0–63.2)	7.54 (0–63.2)	6.37 (0–50.8)
Male (n = 59)	–	8.27 (0–63.2)	–	–
Female (n = 14)	–	2.17 (0–29.6)	–	–
Alcohol (units/wk)	≤ 14	5.96 (0–55.3)	6.39 (0–55.3)	5.58 (0–44.4)
Male (n = 59)	–	7.24 (0–55.3)	–	–
Female (n = 14)	–	1.78 (0–25.9)	–	–
Salt (g/d)	6.0	6.71 ± 2.08	5.44 ± 1.82	7.26 ± 1.96
Male (n = 59)	–	6.98 ± 2.11	–	–
Female (n = 14)	–	5.57 ± 1.58	–	–
Fat (%/d)^d^	≤ 35	33.1 ± 5.53	31.2 ± 5.14	33.9 ± 5.55
Male (n = 59)	–	32.8 ± 4.45	–	–
Female (n = 14)	–	34.3 ± 8.91	–	–
Saturated fat (%/d)^d^	≤ 11	12.3 ± 2.56	11.8 ± 3.05	12.5 ± 2.32
Male (n = 59)	–	12.2 ± 2.35	–	–
Female (n = 14)	–	12.7 ± 3.41	–	–
Protein (%/d)^d^	–	17.4 ± 3.64	17.8 ± 4.55	17.2 ± 3.21
Male (n = 59)	–	17.5 ± 3.78	–	–
Female (n = 14)	–	17.1 ± 3.08	–	–
Carbohydrate (%/d)^d^	≥ 50	44.7 ± 6.99	45.2 ± 7.56	44.4 ± 6.80
Male (n = 59)	–	44.7 ± 5.91	–	–
Female (n = 14)	–	44.7 ± 10.7	–	–
Fibre (%/d)^d^	–	1.36 ± 0.51	1.75 ± 0.60	1.20 ± 0.35
Male (n = 59)	–	1.33 ± 0.51	–	–
Female (n = 14)	–	1.51 ± 0.49	–	–
Alcohol (%/d)^d^	≤ 5	1.65 (0–20.1)	1.74 (0–20.1)	1.47 (0–11.8)
Male (n = 59)	–	2.18 (0–20.1)	–	–
Female (n = 14)	–	0.85 (0–8.16)	–	–

Values represent mean ± SD and median (min–max)

BMR, basal metabolic rate

^a^Dietary Reference Values (DRVs) derived from Public Health England (2016)

^b^Data by gender for Prison officers (M = 15, F = 7) and Firefighters (M = 44, F = 7) not presented due to small sample sizes

^c^Calculated from Schofield Equation

^d^Expressed as percentage of total energy intake

Over 7 days the prison officers spent an average (± SD) total of 40 ± 17 h on-shift and 61 ± 21 h off-shift (4.59 ± 1.74 days out of 7 contained a shift), while firefighters spent average (± SD) of 25 ± 4 h on-shift and 82 ± 9 h off-shift (3.02 ± 0.14 days out of 7 contained a shift). On-shift and off-shift hours accounted for 60% of the total hours of the week that included sleep (sum of all 24 h of the seven days). In prison officers, mean daily intake of energy and sugar was significantly higher when off-shift compared with on-shift (*p* = 0.001 and *p* = 0.002, respectively) (Table 4).

**Table 4:** Comparison of daily energy and nutrient intake during off-shift and on-shift periods

	Total population (n = 73)			Prison officers (n = 22)			Firefighters (n = 51)		
	Off-shift	On-shift	P*	Off-shift	On-shift	P*	Off-shift	On-shift	P*
Energy (kcals)	2,260 ± 620	2,010 ± 873	**0.007**	1,870 ± 378	1,200 ± 820	**0.001**	2,430 ± 630	2,350 ± 621	0.422
Sugars (g)	83.1 ± 29.0	77.5 ± 40.7	0.143	71.5 ± 26.8	48.7 ± 35.3	**0.002**	88.1 ± 28.8	90.0 ± 36.5	0.656
Fibre g	15.8 ± 6.25	13.9 ± 6.92	0.031	16.2 ± 6.26	12.1 ± 8.38	0.029	15.6 ± 6.30	14.7 ± 6.11	0.343
Salt (g)	6.96 ± 2.45	6.06 ± 3.37	0.028	5.75 ± 1.82	4.02 ± 3.49	0.030	7.48 ± 2.52	6.94 ± 2.93	0.257
Fat (%)^a^	33.7 ± 6.06	35.3 ± 8.00	0.073	32.3 ± 6.09	32.7 ± 8.39	0.816	34.3 ± 6.01	36.4 ± 7.64	0.040
Saturated fat (%)^a^	12.2 ± 2.68	13.2 ± 4.60	0.068	11.7 ± 2.94	11.7 ± 3.81	0.982	12.4 ± 2.56	13.9 ± 4.79	0.033
Protein (%)^a^	17.8 ± 3.59	18.3 ± 4.95	0.299	18.1 ± 4.62	19.2 ± 6.61	0.263	17.7 ± 3.08	17.9 ± 4.06	0.634
Carbohydrate (%)^a^	43.8 ± 7.47	44.6 ± 8.86	0.417	44.7 ± 8.00	45.3 ± 10.1	0.759	43.4 ± 7.27	44.2 ± 8.35	0.439
Fibre (%)^a^	1.38 ± 0.57	1.43 ± 0.67	0.479	1.68 ± 0.62	1.91 ± 0.88	0.134	1.25 ± 0.49	1.25 ± 0.43	0.746
Alcohol (%)^a^	1.66 (0–14.5)	0 (0–10.9)	** < **0.001	2.19 (0–11.6)	0 (0–10.9)	0.019	1.66 (0–14.5)	0 (0–6.26)	** < **0.001

Values represent mean ± SD and median (min–max)

^a^Expressed as percentage of total energy intake

^*^p-values relating to the differences between off-shift and on-shift as measured by paired t-test and Wilcoxon test, respectively

The Benjamini–Hochberg procedure for multiple testing of many p-values indicated that only 0.013 and less should be considered significant

**Fig. 1:**
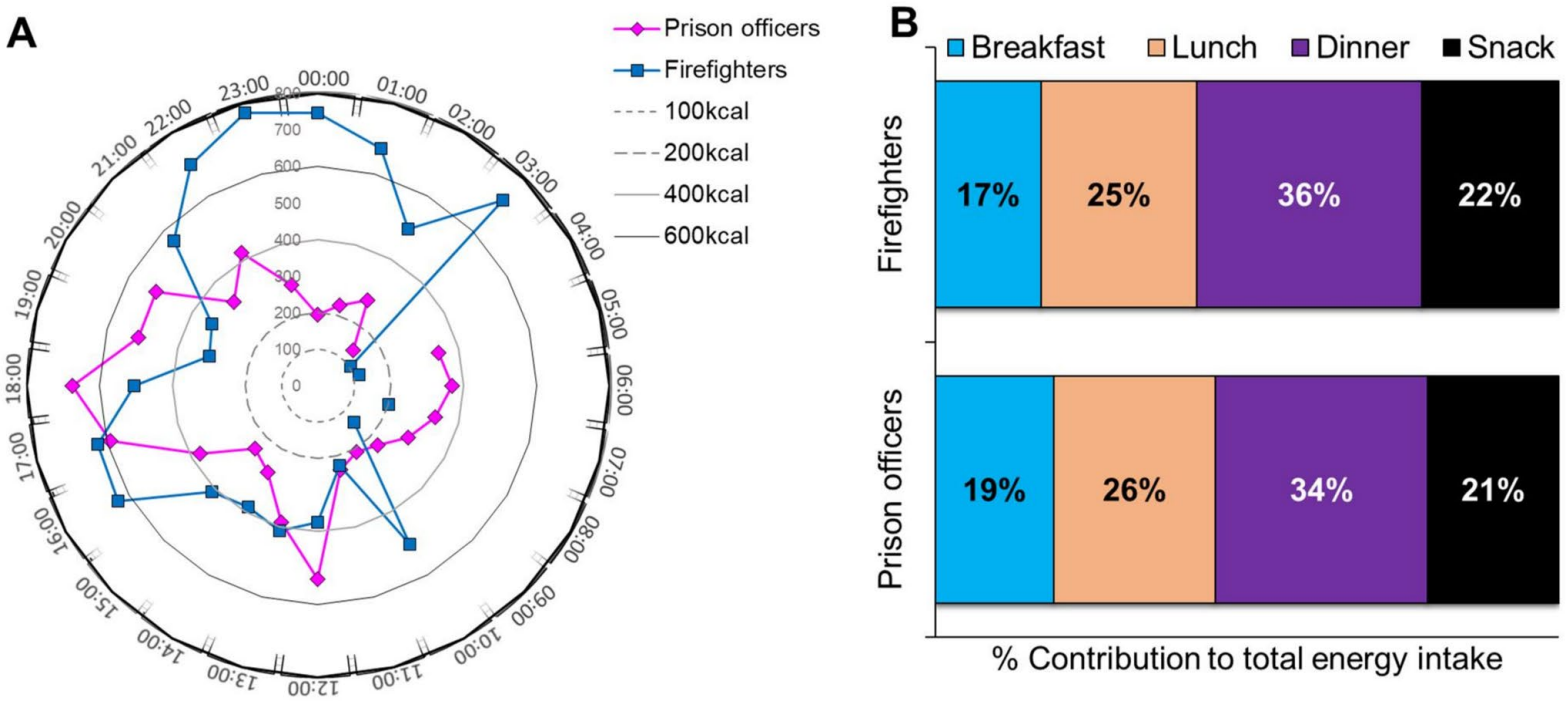
**A** Hourly average energy intake around a 24-hour clock for prison officers and firefighters, using thresholds of 100, 200, 400 and 600 kcal per hourly intake, as assessed by food diaries. **B** Percentage contribution of daily eating occasions to the average total energy intake per day, as assessed by food diaries

Prison officers had peak energy intakes around noon and between 1700 and 2000 h, while the firefighters had peak energy intakes at 1000 h, between 1600 and 1800 h, later at night between 2200 h to midnight and around 0300 h. Generally, energy intake in firefighters was higher during evening hours (Fig. 1A). For both prison officers and firefighters, dinner represented the highest percentage contribution to total energy intake in a day (34% and 36% respectively), while breakfast represented the lowest percentage contribution to total energy intake (19% and 17% respectively; Fig. 1B). These increased eating episodes for the firefighters could correspond hunger after being active during a call, or habitual snacking during downtime. There was a significant association between the shift type and frequency of eating occasions. The frequency of all eating occasions was significantly lower when on-shift than when off-shift, in the total study population and in firefighters (*p* < 0.001), and for breakfast and dinner occasions in prison officers (*p* < 0.001) (Table 5).

**Table 5:** Average frequency of eating occasions (breakfast, lunch, dinner, and snack) during off-shift and on-shift periods

	Total population (n = 73)			Prison officers (n = 22)			Firefighters (n = 51)		
Eating occasions^a^	Off-shift	On-shift	P*	Off-shift	On-shift	P*	Off-shift	On-shift	P*
Breakfast	6 (0–7)	1 (0–5)	** < **0.001	6 (0–7)	0 (0–5)	** < **0.001	6 (1–6)	1 (1–3)	** < **0.001
Lunch	4 (0–6)	2 (0–7)	** < **0.001	2 (0–6)	3 (0–7)	0.083	5 (0–6)	2 (0–3)	** < **0.001
Dinner	5 (2–7)	2 (0–5)	** < **0.001	5 (2–7)	2 (0–5)	** < **0.001	5 (3–6)	2 (0–3)	** < **0.001
Snack	10 (0–50)	4 (0–22)	** < **0.001	7 (0–50)	4 (0–22)	0.244	12 (2–30)	4 (0–14)	** < **0.001

Values represent median (min–max)

^a^Expressed as a frequency of eating occasions across seven days

^*^p-values relating to the differences between off-shift and on-shift as measured using Wilcoxon test

Stress scores were significantly higher during shift days (on-shift days) than on non-shift days (off-shift days) for prison officers and firefighters *p* = 0.007 and *p* < 0.001 respectively. In both prison officers and firefighters, sleep duration per day was significantly longer during non-shift days compared to shift days (*p* < 0.001) (Table 6). Stress did not have significant relationship with diet (Table 7). Being a prison officer or firefighter was the only significant predictor of sleep duration (*β* = − 13.24, *t* = − 4.885, *p* < 0.001), with prison officers sleeping on average 13.2 min per day less than firefighters. These results suggest that occupational factors may influence sleep more than individual anthropometric or demographic variables (Table 8).

**Table 6:** Stress score and sleep duration stratified by shift versus non-shift days across groups

	Total population (n = 73)			Prison officers (n = 22)			Firefighters (n = 51)		
	Off-shift days	On-shift days	P*	Off-shift days	On-shift days	P*	Off-shift days	On-shift days	P*
Stress score^a^	11.4 (0–59)	20.2 (0–71.4)	**<**0.001	12.9 (1–48)	23.5 (0.83–71.4)	0.007	7.75 (0–59)	19.8 (0–56.3)	**<**0.001
Sleep duration (hours)^b^	9.56 ± 1.81	7.69 ± 1.40	**<**0.001	9.13 ± 1.59	7.12 ± 1.03	**<**0.001	9.71 ± 1.88	7.90 ± 1.46	**<**0.001
Sleep duration (hours)^c^	7.81 ± 1.16	6.73 ± 1.20	**<**0.001	8.10 ± 1.37	7.41 ± 0.94	0.032	7.70 ± 1.05	6.46 ± 1.19	**<**0.001

Values represent mean ± SD and median (min–max)

^a^Stress score obtained from averaged Visual Analogue Scale per day

^b^Sleep duration obtained from the averaged actigraph data

^c^Sleep duration obtained from Daily Hassles questionnaire

^*^p-values relating to the differences between off-shift days and on-shift days as measured by paired t-test and Wilcoxon test, respectively

The Benjamini–Hochberg procedure for multiple testing of many p-values indicated that only 0.013 and less should be considered significant

**Table 7:** Relationship between stress and nutrient intake, by shift day

Off-shift						
Nutrient	Total study population (n = 73)		Prison officers (n = 22)		Firefighters (n = 51)	
	r [CI]	*P*	r [CI]	*P*	r [CI]	*P*
Stress^a^						
Energy (kcals)	− 0.167 [− 0.383, 0.068]	0.165	0.123 [− 0.326, 0.527]	0.606	− 0.290^*^ [− 0.521, − 0.018]	0.039
Sugars (g)	− 0.069 [− 0.296, 0.165]	0.567	− 0.329 [− 0.666, 0.120]	0.157	0.010 [− 0.263, 0.282]	0.943
Salt (g)	− 0.138 [− 0.358, 0.096]	0.250	0.335 [− 0.113, 0.670]	0.149	− 0.275 [− 0.510, − 0.003]	0.051
Fat (%)^b^	− 0.019 [− 0.250, 0.213]	0.872	0.203 [− 0.251, 0.583]	0.392	− 0.104 [− 0.367, 0.173]	0.466
Saturated fat (%)^b^	− 0.171 [− 0.387, 0.063]	0.154	0.045 [− 0.394, 0.468]	0.851	− 0.280^*^ [− 0.513, − 0.007]	0.047
Protein (%)^b^	0.151 [− 0.083, 0.370]	0.207	0.363 [− 0.081, 0.687]	0.116	0.076 [− 0.201, 0.342]	0.595
Carbohydrate (%)^b^	− 0.082 [− 0.308, 0.153]	0.497	− 0.518^*^ [− 0.776, − 0.111]	0.019	0.086 [− 0.191, 0.351]	0.548
Fibre (%)^b^	0.066 [− 0.168, 0.293]	0.586	− 0.234 [− 0.605, 0.220]	0.320	0.272 [− 0.001, 0.507]	0.054
Alcohol (%)^b^	0.023 [− 0.210, 0.254]	0.848	0.212 [− 0.242, 0.590]	0.370	− 0.060 [− 0.328, 0.216]	0.676

On-shift						
Nutrient	Total study population (n = 73)		Prison officers (n = 22)		Firefighters (n = 51)	
	r [CI]	*P*	r [CI]	*P*	r [CI]	*P*
Stress^a^						
Energy (kcals)	− 0.083 [− 0.306, 0.148]	0.485	− 0.216 [− 0.577, 0.215]	0.334	− 0.000 [− 0.273, 0.273]	0.998
Sugars (g)	0.020 [− 0.210, 0.247]	0.869	0.046 [− 0.374, 0.449]	0.840	0.040 [− 0.236, 0.309]	0.782
Salt (g)	0.012 [− 0.217, 0.240]	0.918	− 0.128 [− 0.513, 0.300]	0.572	0.122 [− 0.156, 0.382]	0.395
Fat (%)^b^	− 0.030 [− 0.257, 0.200]	0.801	− 0.079 [− 0.476, 0.344]	0.726	0.018 [− 0.256, 0.289]	0.901
Saturated fat (%)^b^	− 0.231^*^ [− 0.437, − 0.003]	0.049	− 0.095 [− 0.488, 0.330]	0.675	− 0.315^*^ [− 0.541, − 0.046]	0.024
Protein (%)^b^	0.029 [− 0.201, 0.256]	0.810	0.027 [− 0.390, 0.434]	0.905	0.021 [− 0.253, 0.292]	0.884
Carbohydrate (%)^b^	0.006 [− 0.223, 0.234]	0.960	0.147 [− 0.283, 0.527]	0.514	− 0.058 [− 0.326, 0.219]	0.687
Fibre (%)^b^	0.058 [− 0.173, 0.283]	0.624	0.025 [− 0.391, 0.433]	0.913	0.046 [− 0.230, 0.315]	0.748
Alcohol (%)^b^	0.000 [− 0.228, 0.229]	1.000	− 0.158 [− 0.535, 0.272]	0.483	0.096 [− 0.181, 0.360]	0.501

r = Pearson correlation coefficient. CI = 95% confidence intervals. P = *p*-value (^*^ *p* < 0.05, ^**^ *p* < 0.01)

^a^Stress score obtained from the hourly Visual Analogue Scale

^b^Expressed as percentage of total energy intake

The Benjamini–Hochberg procedure for multiple testing of many p-values indicated that none of them were small enough to be considered significant

**Table 8:** Ordinary least squares (OLS regression analysis of sleep duration (hours)^a^ by occupational group, waist circumference, and age

Predictor	Coefficient	Std. Error	t-statistic	*p*-value
Intercept	77.37	10.65	7.262	** < **0.001
Group (P)	− 13.24	2.71	− 4.885	** < **0.001
Waist circumference (cm)	− 0.10	0.13	− 0.767	0.446
Age (years)	− 0.20	0.19	− 1.041	0.302

^a^Sleep duration data obtained from the Actigraph. Predictor: Independent variable included in the regression model. Coefficient: Estimated effect of the predictor on sleep duration (in hours). A negative value indicates a decrease in sleep duration with an increase in the predictor. Std. Error: Standard error of the coefficient estimate, indicating the variability of the estimate. t-statistic: The test statistic used to assess whether the coefficient is significantly different from zero

The reference group for "group (p)" is firefighters. A negative coefficient for prison officers indicates they sleep less than firefighters

## Discussion

The current study highlights that dietary intake for shift workers did not meet all UK dietary recommendations and the erratic eating profile, particularly when off shift, in combination with changing stress through daily hassles when on shift, and an interrupted sleep profile contributes to the risk of poor metabolic health status in these occupational groups.

We report that 77.3% of prison officers and 60.7% of firefighters were classified as overweight or obese; on average, over half of prison officers and firefighters were classified as either overweight or obese, consistent with findings in other studies in prison officers in the US [33, 38] and in Columbia [48], and firefighters in the US [39, 49], Germany [50], Cyprus [51] and Russia [52]. In our study, the prevalence of overweight and obesity in prison officers was higher, but that in firefighters was lower, than the prevalence reported for the general population in the Scottish Health Survey [53].

In terms of diet quantity quality and timing, both prison officers and firefighters had a higher daily intake of saturated fat and salt, and a lower intake of carbohydrate and dietary fibre, than the recommended UK dietary reference values (DRVs). For example, the average dietary fibre was about 15-16 g/day, which is far below the UK recommendations of 30 g/day. This deficit is clinically significant, as inadequate fibre intake has been identified as an independent risk factor for chronic diseases including type 2 diabetes mellitus, cardiovascular illnesses and colorectal cancer. Further, a low-fibre, high-fat diet (often termed a Western diet) can negatively affect the gut microbiome, which is closely linked to metabolic health and circadian regulation. This type of diet acts on the gut-liver axis, promoting obesity-linked, low-grade inflammation. Evidence indicates that higher fibre intake reduces all-cause and cardiovascular mortality risk [54, 55]. Notably, dinner was the largest contributor to the daily energy intake with a substantial proportion of the calories consumed late in the day. This pattern misaligns with diurnal rhythms, which favour higher insulin sensitivity and glucose metabolism earlier in the day [56]. Late-night eating has been linked to impaired glycemic control, increased adiposity, and elevated cardiometabolic risk [18, 57]. Therefore, these findings suggest that meal timing may represent an additional mechanism contributing to chronic disease risk in these occupational groups. Interventions should therefore consider strategies that promote earlier energy consumption and reduce reliance on large evening meals.

We found from cross-sectional analysis that shift type appeared to influence eating occasions, with a lower frequency of breakfast and dinner occasions in prison officers when on-shift, and a lower frequency of all eating occasions in the total study population and in firefighters, when on-shift than when off-shift. Also, intake of energy and sugar was significantly higher when off-shift in prison officers. However, interpretation of these differences requires caution as higher intake on off-shift days may reflect reporting bias, since participants may find it easier to accurately record intake during less stressful, non-working days. Under-reporting during busy shifts is a well-recognised limitation in dietary assessment, particularly in occupational groups, and can significantly distort observed associations [58]. The EI:RMR ratio reported was 1.04 and this confirms significant under-reporting, at a level that is deemed below survival for active adults. This is unlikely to reflect caloric restriction, rather, an artefact of the method. As these associations are from correlation analysis, these conclusions should be interpreted with caution, and further investigated, as correlation does not give causation.

Observed patterns of poor diet quality and misaligned meal timing highlight the need for targeted interventions among prison officers and firefighters. Advance meal preparation can reduce dependence on convenience foods during extended shifts, while nutrient-dense snacks such as fruit, nuts, yogurt, and whole-grain options may improve fibre and protein intake, satiety, and energy stability. Additionally, meal timing strategies that prioritise substantial meals earlier in the day and lighter meals later, particularly during night shifts, could better align with diurnal rhythms and support metabolic health [59]. There is a diurnal response in glucose tolerance, in that insulin in more sensitive in the morning period, and less sensitive in the evening period. When consuming the largest meal of the day in the evening, this coincides with the natural decline in insulin sensitivity, which can further exacerbate metabolic health risk. Further, there is evidence of chronotype is independently associated with abdominal obesity and visceral fat, underlining the potential implications of the individual circadian typology on abdominal obesity. The current study did not measure chronotype, which is a person's natural inclination with regard to the times of day when they prefer to sleep or when they are most alert or energetic. Evening type people are usually more active and efficient in the last part of the day, and this has been associated with a health-impairing style, resulting in a higher risk of obesity and cardiometabolic diseases than morning type. The science of chrono-nutrition or timing of eating with chrono-type is relatively new, and more focus on the role of shift work is needed for targeted improvements in health. Integrating these approaches into workplace wellness programs and institutional catering policies may enhance diet quality and mitigate chronic disease risk in these population.

Shift work is known to detrimentally affect important health outcomes such as body weight, stress and sleeping patterns [60]. In previous studies, shift work has also been linked to higher stress levels in prison officers [32, 38] and firefighters [61]. Various assessment tools have been used to evaluate stress in shift workers; we used the Daily Hassles tool, which provides insights into the sources of stress [43], a Visual Analogue Scale to measure temporal changes of stress prospectively [42], and the DASS-_21_ questionnaire [62] to provide a retrospective assessment of stress. Different assessment tools will give differential insights into stressors and levels of stress, as indicated in our current study and therefore, outcomes between tests, and between studies, may be difficult to compare. In this study, both prison officers and firefighters had stress scores within the normal range when using the DASS-_21_ tool, whereas an Iranian study recorded high stress scores for prison officers [32], which may reflect a harsher infrastructure or working environment for these officers. The stressors observed in our study were similar to those observed in Korean firefighters, which recorded a higher score for job stressors as a source of stress [63]. This study also revealed that stress levels varied according to shift as results averaged from the Visual Analogue Scale indicated that both groups had significant higher score of stress during shift days than on non-shift days.

Shift work has been reported to influence sleep and performance of the work force [15, 63], and shorter sleep duration has been linked to an increased risk of cardiovascular disease [16] and of overall burnout [9]. In our study, sleep duration in both prison officer may reflect reporting bias and firefighters was within recommendations (7–9 h) [64]. Although both professions are run under a shift system, there are slight differences in shift schedules for prison officers with those of firefighters, which may contribute to the difference in sleep duration [65, 66]. Average sleep duration in firefighters in our study was very similar to that observed in other studies, especially when firefighters had no fire call-out [16, 67]. Our study also revealed that in both groups, participants slept more during days when they were not on shift. US studies have reported lower than recommended sleep duration in firefighters [68, 69] and in prison officers [33]. A notable finding was the discrepancy between actigraphy and self-reported sleep duration, consistent with previous studies in shift-working populations. For example, a study of firefighters found that participants had wide variability in total sleep time estimates compared to actigraphy [70]. Such differences may reflect cognitive biases, or poor recall of sleep duration. Subjective measures often capture perceived restfulness, while actigraphy may misinterpret inactivity as sleep [71]. These findings highlight the importance of using both objective and subjective tools to assess sleep in shift-working populations like firefighters and prison officers. Interestingly, job satisfaction and co-worker support have been linked to improved sleep duration and better sleep quality in prison officers [33], and these factors may therefore be an important factor to take into account when comparing the outcomes in the different studies.

Detrimental health outcomes in shift workers may be indirectly caused by changes in conventional eating times and unhealthy dietary patterns, resulting in excessive intakes in calories or nutrients, such as saturated fat, sugar and salt [10, 19]. In our study, the intake of the macronutrients was generally within UK diet recommendations. Both prison officers and firefighters had a higher intake of saturated fat and salt than recommended, but levels were lower than those reported for the general population in two recent diet and nutrition surveys [72, 73]. Previous studies in prison workers have reported that time constraints led prison officers to eat whilst on the run, and relying on food vending machines or the consumption of unhealthy snacks that could be eaten quickly [33, 38]. However, in both prison officers and firefighters, meals rather than snacks contributed the highest proportion of total energy intake, which is in agreement with previous studies in police officers [19] and in garbage collectors [74]. However, we also observed that in prison officers, but especially in firefighters, energy intake peaked later in the day, which was also observed in previous studies [19, 20, 75]. Rotating shift work may lead to irregular eating times [17, 75] and to interruption of the diurnal cycle [3]. Outcomes from clinical studies suggest that irregular timing of dietary intake affects glucose metabolism, alters blood pressure, interferes with hormone regulation (leptin and cortisol) and cause heart rate variability [76, 77]. Both observational and controlled studies have shown that eating late is linked to an increased risk of overweight and obesity [22, 23], and a shorter time- of restricted eating led to better health outcomes and an improved Mediterranean diet score in firefighters [78]. Furthermore, a lower frequency of breakfasts occasions, as observed in both prison officers and firefighters who were on-shift, as well as in other studies in shift workers [75], has been associated with a lower diet quality [18, 26], increased adiposity, as well as with conditions like type 2 diabetes mellitus [79] and cardiovascular disease [18].

In this study, the observed increase in stress and shorter sleep duration on shift days may have likely contributed to the poor dietary patterns identified. Both stress and sleep restriction are well-established physiological drivers of unhealthy food choices. Elevated stress can promote emotional eating and preference for energy-dense, high-fat, and high-sugar foods through activation of the hypothalamic–pituitary–adrenal (HPA) axis and reward pathways [80]. Stress also influences appetite-regulating hormones, with ghrelin (orexigenic) increasing and leptin (anorexigenic) decreasing during stressful conditions, thereby stimulating hunger and energy intake [81, 82]. Similarly, sleep deprivation disrupts hormonal balance, elevating ghrelin and reducing leptin, which increases appetite and preference for high-calorie foods [83, 84]. These mechanisms provide a compelling explanation for the reliance on convenience foods and late-night eating observed among prison officers and firefighters. Integrated interventions should therefore address not only dietary behaviours but also stress management and sleep hygiene to improve overall health outcomes.

In our study, prison officers recorded a low ratio of energy intake to basal metabolic rate (BMR) [85]. This suggested that prison officers might have underreported their dietary intake, and this could have affected our findings. Being overweight or obese, and having busy working schedules, are known factors related to underreporting energy intake when collecting dietary information [58]. Our findings on association between stress and nutrient intake found few weak associations. However, none of these were strongly significant (none are < 0.01) hence we may conclude that there is no clear evidence of associations between stress and nutrient intake. These results differ with other studies that have reported that stress was associated with an increased intake of foods that are high in fat, saturated fat and energy mostly when on shift [8, 86, 87]. Nevertheless, findings may differ due to the assessment tools, type of occupation or the sample size used. Finally, in the total population of prison officers and firefighters, we observed an inverse relationship between sleep duration and trunk body fat and visceral fat. Similarly, another study in firefighters found that those having less than 6 h sleep per night had higher weight, waist circumference, body fat and body mass index [16]. Conversely, both shift work and body composition are established risk factors for sleep disorders such as Obstructive Sleep Apnoea (OSA). Although OSA was not assessed in this study, its high prevalence among firefighters and law enforcement officers suggests it may be a relevant confounder [88, 89].

This study is one of the few studies exploring how shift work schedules, stress, and sleep affect the quantity and quality of dietary intake, and eating times, in prison officers and firefighters in the UK. The strengths of this study include the collection of comprehensive information on body composition, shift work and sleep, and application of 7-day weighed food diaries and scales to collect prospective information on dietary intake, dietary patterns, frequencies and nature of eating occasions.

This study had several limitations that should be considered when interpreting the findings. For example, the cross-sectional design limits the ability to establish causal relationships between shift work, dietary intake, and health outcomes. Longitudinal research has demonstrated that dietary behaviours among shift workers can change over time, influenced by factors such as adaptation to shift schedules and cumulative fatigue. The absence of data on shift work experience is a key limitation, as, it is not possible to account for the potential influence of cumulative exposure to shift work. This may introduce bias due to the "healthy worker survivor effect," whereby individuals who are more resilient to the demands of shift work remain employed, potentially leading to an underestimation of the true associations. Additionally, this study recruited a relatively low number of prison officers within this cohort, which may have limited the statistical power to find significantly different dietary patterns, and only 19% were female, which may give a bias of gender representation. Furthermore, we performed several statistical comparisons and calculated multiple p-values, which were adjusted for multiple testing using the Benjamini–Hochberg procedure. Methods such as Bonferroni would have made unsuitable assumptions such as independence of tests, whereas variables in our study were clearly correlated. This does mean that some of the marginally significant p-values can be considered unreliable, while the more strongly significant p-values would remain significant after any adjustment. We therefore focused in our discussion on these stronger results.

These results support future research to explore workplace nutrition policies, including the impact of healthier canteen menus, reduced access to high-fat and high-salt foods, and adjustments to meal timing, may also offer practical avenues for intervention. Longitudinal and intervention studies are important to track health changes over time and evaluate the effectiveness of strategies aimed at improving diet, physical activity, and weight management in these occupational groups, while both on-shift and off-shift. Moreover, future work should consider broader determinants of health, such as sedentary behaviour, workload, coping strategies for stress, and recovery periods, in order to develop a more holistic understanding of the challenges faced by shift workers. Ultimately, these findings could support the creation of tailored public health guidelines and workplace policies that address the unique needs of employees working non-standard hours.

In conclusion, we showed that especially prison officers had a higher prevalence of overweight and obesity compared with the general population, but levels of stress and sleep duration were within the normal range. This group of shift workers had adapted dietary patterns which would increase their risk of metabolic disease, including an increased intake of saturated fat and salt, and an increased intake of energy in the evening hours. Besides dietary patterns, sleep and stress, this study may suggest that the prevalence of overweight and obesity could be influenced by other risk factors that were not fully explored, such as physical activity.

## Supplementary Information

Below is the link to the electronic supplementary material.

Supplementary file1 (DOCX 69 kb)

## Data Availability

Anonymised data is available as an electronic supplementary file or by contacting the corresponding author.
