# Supplementary material for: Association of shift work with body weight, stress, sleep, and dietary intake in prison officers and firefighters
Source: Eur J Nutr. 2026 Mar 5;65(3):81. doi: 10.1007/s00394-026-03923-x (PMC12963118; doi:10.1007/s00394-026-03923-x)
Supplement: Supplementary file 1 — Supplementary file1 (DOCX 69 kb) [file 394_2026_3923_MOESM1_ESM.docx]

**Supporting Information**

Online Resource 1. NeuroFAST Study protocol diagram


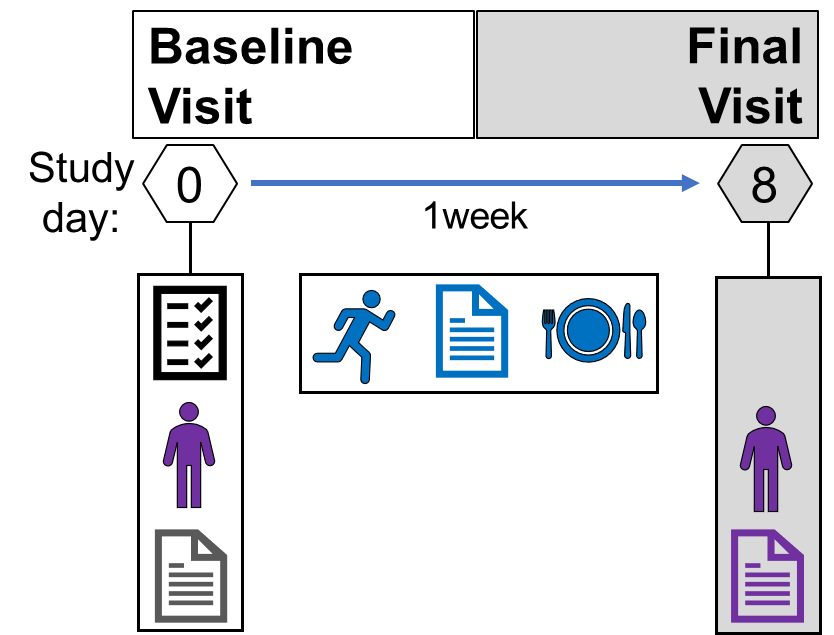


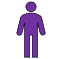
body composition measurements: abdominal fat, height, waist and hip circumferences (Day 0 only) and body weight (Days 0 and 8);
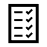
consent paperwork on day 0;
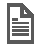
day 0 questionnaires (eating behaviour, personality);
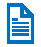
day 1 to 7 questionnaires (daily hassles, appetite and stress);
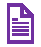
day 8 questionnaire (DASS-21);
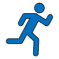
physical activity measurement on days 1 to 7;
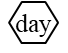
study visit at participant’s workplace;
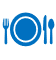
weighed food intake record for 7days.

DASS-21, Depression, Anxiety and Stress Scale questionnaire; NeuroFAST, neurobiology of food addiction and stress
